# Risk factors of bovine tuberculosis in cattle in rural livestock production systems of Ethiopia

**DOI:** 10.1016/j.prevetmed.2009.02.006

**Published:** 2009-06-01

**Authors:** Rea Tschopp, Esther Schelling, Jan Hattendorf, Abraham Aseffa, Jakob Zinsstag

**Affiliations:** a Swiss Tropical Institute, PO Box, CH-4002 Basel, Switzerland; b Armauer Hansen Research Institute (AHRI/ALERT), PO Box 1005, Addis Ababa, Ethiopia

**Keywords:** Ethiopia, Bovine tuberculosis, *Mycobacterium bovis*, Prevalence, Cattle, Risk factors

## Abstract

This study shows a representative stratified cluster sample survey of the prevalence of comparative intradermal tuberculin test in cattle from four regions in Ethiopia. Using a cut-off for positivity of 2 mm, it assesses possible risk factors for tuberculin-positive reaction in cattle. Seventy-three villages in 24 kebeles (administrative units) were randomly selected, from which 2216 cattle from 780 owners were tested. In addition, 450 of these cattle owners were interviewed for risk factor assessment. Ninety-nine percent of the tested cattle in this rural livestock production system were traditional zebus. The individual overall prevalence of cattle bovine tuberculosis (BTB)e was 3%, with the highest found in Meskan Mareko, in Central Ethiopia (7.9%) and the lowest in Woldia, in the North East edge of the Rift Valley (1.2%). Generalised Linear Mixed Models (GLMM) with random effect on kebeles was used to analyse risk factors of cattle reactors and human tuberculosis (TB) infection. Purchase of cattle and presence of other livestock in the herd were statistically significant, with OR: 1.7, *p*-values of 0.03 and OR: 2, *p* = 0.05, respectively. Family members diagnosed with TB or showing clinical signs of extra-pulmonary TB (EPTB) were reported in 86 households (19%). None of the assessed potential risk factors of disease transmission between cattle and human (food consumption, livestock husbandry and presence of BTB-positive cattle) were statistically significant.

Bovine tuberculosis (BTB) is an infectious disease caused by *Mycobacterium bovis*, a member of the *Mycobacterium tuberculosis* complex (MTC), which also comprises the closely related *M. tuberculosis*, the major causative agent of human tuberculosis (TB) (Van Soolingen et al., 1994). Around 9 million new cases of TB and 2 million deaths are reported worldwide annually (CDC, 2007), with sub-Saharan Africa displaying the highest annual risk of infection with TB, probably catalysed by the HIV/AIDS pandemic (Corbett et al., 2003). It has been estimated that *M. bovis* accounts globally for 3.1% of all human TB cases (2.1% of all pulmonary and 9.4% of all extra-pulmonary TB (EPTB) cases) (Cosivi et al., 1998). However, the extent of *M. bovis* involvement in the global TB burden in Africa is still largely unknown. This can be partly explained by the fact that in humans, TB due to *M. bovis* is indistinguishable from that due to *M. tuberculosis* in terms of clinical signs, radiological and pathological features (Grange, 2001). In addition, most laboratories in sub-Saharan Africa do not have the capability to differentiate *M. bovis* from *M. tuberculosis (*Ayele et al., 2004*).*

The epidemiology of *M. bovis* was described by numerous authors in extensive detailed reviews; however, they tend to focus mainly on experiences from industrialised countries, where control and/or eradication programmes have been implemented since a long time (O’Reilly and Daborn, 1995; Cosivi et al., 1998; Pollock and Neill, 2002; Wedlock et al., 2002; Phillips et al., 2003; Neill et al., 2005). Although cattle are considered to be the main hosts of *M. bovis*, the disease has been reported in many other species, including humans, other domesticated animals and wildlife (De Lisle et al., 2002). Infectious animals shed *M. bovis* via milk, saliva, faeces/urine and discharging lesions (Phillips et al., 2003). It is generally accepted that human beings get infected either by inhalation of bacteria-containing dust-particles and aerosols shed by infected animals or by ingestion of contaminated animal products (e.g., raw milk) (Cosivi et al., 1998). The main route of infection in cattle is through aerosol exposure, facilitated by close contact between animals (Neill et al., 1991). In cattle, ingestion of contaminated products (e.g., pasture and water) is generally considered to be a secondary, less important route of transmission (Menzies and Neill, 2000). A high prevalence of *M. bovis* was found in a recent abattoir survey in Chad in culture-positive mammary glands and in young tuberculin-positive animals (Müller B, personal communication). Jha et al. (2007) isolated *M. bovis* in milk and faeces from milking buffaloes and cattle in Nepal. This indicates that transmission to young animals by milk should not be neglected. Recent publications from Africa also suggest that ingestion of *M. bovis* might be an important mode of disease transmission in cattle, since mesenteric lymph nodes were shown to be more affected than mediastinal lymph nodes (Cleaveland et al., 2007, Ameni et al., 2007). Therefore, contaminated environment might also play a bigger role in the epidemiology of BTB than assumed until now, thus showing that our understanding of the epidemiology of *M. bovis* in sub-Saharan Africa is still limited.

Indeed, little information on risk factors of disease transmission to cattle, between cattle and from cattle to humans is available from the African context; most information is extrapolated from experiences in the industrialised world. For Africa, the most comprehensive epidemiological studies done so far have been in Tanzania (Kazwala et al., 2001; Cleaveland et al., 2007; Mfinanga et al., 2004) and Uganda (Oloya et al., 2007). However, despite the paucity of information, it is generally accepted that besides causing major economic losses, BTB also poses a serious zoonotic threat in Africa (Ayele et al., 2004).

BTB has been shown to be endemic in cattle from Ethiopia. However, most of the published data were obtained from central Ethiopia (Ameni et al., 2003, 2007; Asseged et al., 2004; Teklul et al., 2004).

In this article, we attempt to mainly assess possible risk factors for BTB in cattle and humans in rural Ethiopia. We also present briefly the results of a representative multistage cluster sample survey on BTB prevalence in cattle from four regions of Ethiopia for better understanding of the context.

## 1 Materials and methods

### 1.1 Study sites

The cross-sectional study was carried out between November 2006 and May 2007 in the frame of a North–South BTB research collaboration, in three out of the seven regional zones of Ethiopia (Oromia, Amhara, and Southern Nations, Nationalities and People Region (SNNPR)) between the latitudes of 5.1°N and 11.5°N and the longitudes of 36.1°E and 40.1°E. Within these regions, six woredas (districts) (Woldia (Northern highlands), Meskanena Mareko (Rift Valley), Bako Gazer (Southern middle lands), Dinsho, Robe and Goro) were selected according to the requirements of the study as a whole (e.g., presence of abattoirs, hospitals and wildlife). The latter three woredas were combined into one study area, the Bale Mountains, which is a highland area adjacent to a national park. Altitude of the study sites varied between 1300 and 4200 m above sea level. Although belonging to different ethnic groups with different culture and religion, all farmers were sedentary small holders with similar mixed livestock-crop farming system.

## 2 Tuberculin survey in cattle

### 2.1 Sampling of cattle

Cattle herds were selected by a stratified cluster sampling proportional to the size of the cattle population, in which the unit village was considered as a cluster. Sample size was calculated using the formula described by Bennett et al. (1991).

The standard error *s.e.*_*x*_ which measures the precision of our estimate is given by (1):

(1) $$s.e._{x}=\sqrt{\frac{pqD}{n}}=\sqrt{\frac{pqD}{cb}}$$

*Roh* (*ρ*) describes the rate of homogeneity, thus the variability is given by (2):

(2) $$\rho =\frac{\left(\text{Within}\;\text{Herd}\;\text{Variation}\right)}{\left(\text{Total}\;\text{Variation}\right)}$$

(3) D=1+(b−1)ρ

We take *roh (ρ)* as 0.2, and using the formula we obtain a design effect *D* of 6.8 (3).

Choosing 30 animals per cluster with a disease prevalence of 5% and 17 clusters (total sample size per woreda = 510 animals) gives us an estimate of the standard error (1) or precision of 0.025. The total sample size per woreda is given by *n =* *b* × *c*, thus 510 animals, which gives us a total number of required animals of 2040 for all four woredas. A complete list of kebeles and villages within the kebeles was obtained from each woreda agricultural office. Kebeles within the woreda were selected randomly using random numbers generated in Microsoft Excel^®^; villages were selected randomly and proportionally to their number within a particular kebele. Since cattle of all villagers are kept together during the day, at least on grazing areas and for drinking, each village was considered as one big cattle herd for assessment of tuberculin reactivity status. Thirty animals were selected from a minimum of five and a maximum of 15 owners per village. In general, not all animals per owner were tested since as many owners as possible per village were included in the study, either randomly from a list of all owners (where owners numbered higher than 15) or including the total number of owners gathered at the place where the tuberculin testing was performed (where owner number was less than 15). Animals younger than 6 months, cows at a late stage of pregnancy and clinically sick animals were excluded from the testing. Possible sampling bias was introduced when the owner himself decided which animals fulfilling the inclusion criteria were tested.

Farmers were asked to gather their animals at a certain point in or in the proximity of the village (e.g., communal pasture, water point and middle of the village) for testing and reading. If animals were not at the meeting point during the reading day, a house-to-house visit was conducted. As compensation and incentive for farmer's participation, all tested cattle were de-wormed on the reading day with Albendazol boli (Ashialben 2500, Ashish Life Science PVT, Mumbai, India).

### 2.2 Tuberculin testing of cattle

The same person conducted the entire process of tuberculin testing and reading of the result to avoid bias related to injection and reading technique. The comparative intradermal tuberculin test was conducted in all cattle using both avian and bovine purified protein derivates (PPD) supplied by the Veterinary Laboratories Agency, Weybridge, UK. Intradermal injections of 0.1 ml (2500 IU ml^−1^) bovine PPD and 0.1 ml (2500 IU ml^−1^) avian PPD were administered in two shaved sites, 12 cm apart from each other in the middle neck region, after having recorded skin thickness with a caliper. Skin thickness was measured again at both injection sites after 72 h. The reaction at each site was derived by measuring the difference of the skin thickness before and 72 h after the injection. An animal was considered positive if the bovine minus the avian reaction was greater than 2 mm (Ameni et al., 2008). A village herd was considered positive if it had at least one positive tuberculin reactor.

### 2.3 Interview of cattle owners

Cattle owners were interviewed according to their willingness to participate and after verbal consent on the same day that their cattle were tested for BTB. Interviews were conducted on all sites in Amharic by a trained interviewee. Questionnaires included closed and open questions on livestock husbandry/management and household characteristics, such as herd size and structure, presence of other livestock, vaccination/de-worming of cattle, mixing of cattle and other livestock at night, cattle housing at night, watering and grazing system, reproduction, cattle contact with other cattle herds and purchasing of animals. Furthermore, questions related to human consumption habits, contact between humans and cattle, knowledge of TB and known TB status in the household were also asked. A household was considered positive for TB if at least one member in the last 5 years had been diagnosed with pulmonary TB or showed clinical signs of EPTB (e.g., cervical lymphadenitis). In addition, focus group discussions were conducted in the villages.

Geographic coordinates and altitude were registered at the central point of each village by global positioning system (GPS).

### 2.4 Statistical analysis

Data were double entered in Access, validated with EpiInfo (version 3.3.2) and analysed with the software package STATA/IC 10.1 (StataCorp, Texas, USA). Analysis of potential risk factors for the cattle being positive for BTB and estimation of variance component were performed using generalised linear mixed models with binary outcome and logit link function (GLLAMM add-on). The exploration of the different variance components of each stage of the multistage cluster sampling indicated that the random effect variances were mainly associated with each woreda and kebele. In contrast, the variance components of owner and village level were <0.0001. Therefore, we included in all models the kebele as random and woreda as fixed effects.

## 3 Results

### 3.1 Prevalence of tuberculin-positive cattle

Seventy-three villages in 24 kebeles in the four woredas were selected, of which a total of 2216 cattle from 780 cattle owners were tested for BTB. Ninety-nine percent of the tested animals were traditional zebus, 1% accounted for exotic Holstein breed and cross-breeds (Holstein and zebu). The overall apparent individual animal prevalence of tuberculin reactors was 3.1% (95% CI: 2; 4.8) but varied significantly between the woredas in Meskan Mareko with the highest prevalence and the lowest in Woldia (Table 1). Of the 73 village herds tested, 49 had at least one positive tuberculin reactor, giving an overall herd prevalence of 67%.

### 3.2 Descriptive epidemiology

In this survey, 450 farmers were interviewed. Fifty-six owners (12.4%) had PPD-positive cattle, and among these farmers, 24% reported TB cases in their household. Cattle fodder consisted of 90% pasturing and crop residues after harvest. Fifty-four percent and 31% of the farmers vaccinated their cattle against blackleg and/or pasteurellosis and de-wormed on a regular basis.

Livestock other than cattle were kept in mixed herding systems in 70% of the farms interviewed (31% goats, 45% sheep, 25% donkeys, 9% horses and 2% camels). Sixty-two percent of the cattle herds grazed on communal pasture either full-time or part-time, 81% were watered at the river and 99% of the herds had close contact with other cattle herds the year round during communal grazing and/or watering, veterinary campaigns, communal harvesting–ploughing and/or threshing. Overall, nearly half of the farmers (46%) kept cattle inside the living housing at night. Natural service for cow fertilisation was used in 92% of the farms with 54% of the farmers using a bull from a neighbouring farm for reproduction. Encounters between wildlife and cattle were overall rare (observed and reported by 19% of interviewees with the exception of the Bale mountains national park, where 59% of farmers stated that wildlife share common habitat with cattle. Herding was mainly done by children (37%) or children and men combined (50%). Twelve percent of the herds were looked after by adult men, whereas women were rarely involved in herding (0.4%). Herds were not looked after at all in 0.7% of the interviewed households. In contrast, milking of cows was mainly carried out by women (45.5%) or adult men and women combined (44.5%). In 9.5% of the interviewed households, adult men were milking cows. Children were rarely involved in milking tasks (0.5%). Eighty-one percent of the farmers did not boil the drinking milk and 74% ate raw meat. The farmers’ knowledge of the clinical signs of TB, either in humans or in cattle, was generally high (70%). However, we noticed during focus group discussions that farmers often did not know that the disease could be transmitted from cattle to humans.

### 3.3 Risk factors for positive PPD in cattle

We assessed 23 potential risk factors for BTB positivity in cattle. Variables with more than 10% missing values and variables, which are assumed to be redundant, were excluded from the analysis to ensure sufficient power and to avoid co-linearity in the multivariable model. In case of co-linearity, we included the biologically more plausible variable in the multivariable model. The significant variables resulting from the univariable analysis were purchase of cattle (OR: 1.7, CI OR: 1; 2.9, *p *= 0.04) and the presence of other stock (OR: 2, CI OR: 1; 4, *p *= 0.05) (Table 2).

We excluded the variable ‘presence of sheep’ for the multivariable model due to co-linearity with the variable ‘presence of other stock’. We included following variables in the multivariable analysis according to the criteria mentioned earlier: presence of other stock, purchase of cattle, cattle that were not de-wormed and communal grazing. All these variables showed a higher risk for having positive cattle reactor, although none of them were statistically significant (Table 3).

### 3.4 Potential risk to people

TB (confirmed clinical diagnosis, clinical signs of cervical lymphadenitis) was reported in 86 households (19%). At least 20% of the reported cases were EPTB (cervical lymphadenitis). None of the assessed eight variables were statistically significant in the univariable model for having a TB case in a household (Table 4).

## 4 Discussion

Our study reports a representative estimate of BTB prevalence in rural Ethiopia. The overall prevalence found in our study is consistent with the results found in some African countries. In Tanzania, Shirima et al. (2003) and Cleaveland et al. (2007) found individual cattle prevalence of 1.3% and 0.9%, respectively. Our survey shows apparent prevalence by using a cut-off for skin test reaction of 2 mm as suggested by Ameni et al. (2008), who stated that maximum sensitivity can be achieved in central Ethiopia using a 2 mm cut-off without affecting specificity. However, the prevalence shown in this survey is still low compared to previous results published from central Ethiopia, where prevalences between 7.9% and 11.6% were observed in local zebus, using a 4 mm cut-off (Ameni et al., 2003, 2007). This suggests that BTB epidemiology in rural extensive systems very likely differs from peri-urban livestock-production systems (central Ethiopia) in the country. This could be explained by different husbandry practices and a higher number of cross-breeds and exotic breeds (considered to be more susceptible to BTB) found in peri-urban settings. Bovine TB seems to be endemic and widespread in rural Ethiopia, with 67% of the tested village herds having at least one positive reactor. Regions located in higher altitudes seem to have a lower prevalence (Table 1); however, there was no statistical association in our study. Still, the role of altitude seems worth pursuing in further research, since it has been shown that in human TB, altitude was negatively correlated with prevalence of TB (Vargas et al., 2004; Saito et al., 2006).

Although the situation in the sampled villages is favourable for BTB transmission among animals (very close contact throughout the year in poor ventilated houses, communal grazing/ploughing/threshing/watering, overcrowded pastures), only a few animals per herd were found positive, with an overall individual prevalence of 3%. This indicates a low transmission in the investigated livestock systems which are characterised by small herd size; mostly communal grazing with some crop residue supplementation; if housing, then only at night but not during the day and cattle that are often kept together with small ruminants.

In contrast to Cleaveland et al. (2007) and Ameni et al. (2003), herd size did not play a significant role as risk factor for tuberculin reactivity. This could be explained by the high proportion (81%) of small herds with less than 10 animals in our study, making comparison of different herd size difficult.

There seems to be very little transmission during extensive communal grazing, even on crowded pasture. Similarly, Francis (1947) observed that if young stock grazes with heavily infected older stock, the infection rate would remain low until they are brought back into stabling. This could also indicate that, among others, transmission might be maintained by a few cows shedding mycobacteria in their milk causing early infection of young animals, of which a low proportion themselves become infectious in later stage. Our result suggests that spread of disease may not necessarily be linked with the daily gathering of many animals from different herds at one site, as often suggested in the literature, and definitely needs further investigation, that includes other ways of gathering such as watering of animals as well. We could not do a proper statistical analysis of the effect of different watering sources since only 3% of the animals were not watered at communal sources. Cattle that were not regularly de-wormed were nearly twice at risk for being a reactor, but the result was not statistically significant (OR: 1.9, *p* = 0.1). However, this result suggests that high parasitic loads may decrease the animal resistance and make it more susceptible to BTB.

In our study, and in contrast with the recent findings in Tanzania conducted with similar settings (Cleaveland et al., 2007), keeping cattle inside at night, although increasing the risk for having reactors, was not statistically significant for BTB positivity in cattle (OR: 1.9, *p *= 0.2), despite prevailing poor ventilation and very close animal contact. This could be explained by the low number of animals kept indoors. We did not investigate any direct environmental risk factors. However, recent studies in Tanzania (Cleaveland et al., 2007) and Uganda (Oloya et al., 2007) suggested that flooding may play an important role in disease transmission. Environmental source of infections should be more thoroughly investigated in future research since their importance in the epidemiology of BTB transmission remains elusive.

Animals older than 10 years were at higher risk of infection (OR: 1.9), which is in line with findings by Cleaveland et al. (2007) from Tanzania. Phillips et al. (2002) also suggested that older animals were more susceptible for *M. bovis* infection. However, in our case, the result was not statistically significant (*p* = 0.3). This might have been due to the low number of old animals in our study (*N* = 35; 7%). These findings suggest, nevertheless, that removing old animals from a herd and avoiding purchasing old animals from markets might help decreasing-within-herd prevalence and risk of introduction of infectious animals, respectively. Purchase of animals was significantly associated with BTB positivity (OR: 1.7; *p* = 0.046), suggesting that the disease is likely to be spread regionally by animal movements.

Having livestock other than cattle in the herd increased the risk of positive tuberculin reaction in cattle (OR: 2, *p* = 0.05). Although not statistically proven, keeping sheep in the farm increased the risk of finding positive PPD cattle (*p* = 0.07; OR: 1.7). Nearly half the farmers having stock other than cattle, owned sheep, which are kept in mixed herding with cattle. Bovine TB in sheep is rare but has been nevertheless described in Europe (Malone et al., 2003) and Sudan (Tag el Din and Gamaan, 1982) and should be further investigated, considering the large sheep population in Ethiopia.

The reported human TB in our study comprised all forms of the disease and no differentiation was made between *M. tuberculosis* and *M. bovis*. Considering the high percentage of extrapulmonary cervical lymphadenitis recorded (at least 20% of all reported TB cases in households), it is plausible that *M. bovis* might play a role. Farmer consumption habits (raw milk, raw meat) did not show any statistical significance, as against previous findings in Ethiopia (Ameni et al., 2003). In contrast to Regassa et al. (in press) and Ameni and Erkihun (2007), we did not find any correlation between having PPD-positive cattle and a human case of TB in the household, even though people and cattle often shared the same house. However, statements on the zoonotic potential of BTB require confirmed *M. bovis* cases to address their risk factors specifically. Vice versa, having confirmed TB cases in a household was not associated with cattle reactors (OR: 1.1; *p* = 0.7).

Such high household interviews (450), as conducted in our study, have rarely been conducted in the past. However, considering the very low prevalence of the disease in the country, the power of the study should be further increased in future research. Because of the clustered distribution of livestock, random effects models are more appropriate and risk factor assessments more conservative. Further studies on risk factors of BTB in humans require case–control studies with confirmed *M. bovis* infection. ‘Classical’ risk factors have been investigated to a certain extent in small studies in the Ethiopian Highlands, sometimes showing divergent results. However, in order to embark in a national BTB control programme, thorough knowledge of possible risk factors is an essential prerequisite and should, therefore, also include risk factors associated with environment and milk as well as the role of co-infection in cattle with diseases highly prevalent in some areas (e.g., trypanosomiasis, fasciolosis, chronic contagious bronchopneumonia), nutritional challenges and the genetic role of different cattle breeds to BTB susceptibility.

It appears that the epidemiology of BTB varies not only between different African countries but also between different regions in Ethiopia depending on livestock systems (extensive, intensive), breeds (local, exotic, cross-breed) but also ecological and geographic factors. Further research is needed to better understand BTB transmission in extensive livestock systems of Ethiopia as well as the true potential of zoonotic risk of transmission and finally to address the potential of control options.

## Figures and Tables

**Table 1:** PPD prevalence in cattle in four different woredas using a cut-off of >2 mm (calculated using a logistic regression model with kebele as random effect and woreda as fixed effect).

Woreda	Number kebele	Altitude range (m)	Number village	Number positive village^a^	Total tested animal	PPD-positive reactors	Percentage of reactor animals (prevalence)	95%CI
Meskanena Mareko	5	1800–2170	21	20	590	47	7.9	5.8–10.5
Woldia	6	1460–3500	22	8	629	13	1.2	0.3–3.9
Bako Gazer	7	1330–1640	19	14	542	25	4.3	2.3–7.7
Bale Mountains	5	2120–3500	11	7	455	9	2.0	1.0–3.8

Total	23		73	49	2216	94	3.1	2.0–4.8

a A village is positive if it has at least 1 positive reactor.

**Table 2:** Univariable analysis of risk factors for cattle tuberculin reactor using GLMM models with kebele as random effect.

Risk factor		Proportion% (No/Total)	OR	95% CI for OR	*p*-value
Presence of other stock		70 (313/450)	2	1; 4	0.05
Presence of sheep		45.3 (204/450)	1.7	1; 3	0.07
Purchase of cattle		38 (172/450)	1.7	1; 2.9	0.04
Communal grazing		62 (265/428)	1.5	0.9; 2.6	0.1
Not de-wormed cattle		24.3 (109/449)	1.8	0.9; 3.8	0.1
Presence of old animals (>10 years)		7 (35/450)	1.5	0.7; 3.3	0.3

Cattle housing night	Base: free-roaming				
	Outside shed	11 (48/449)	1.4	0.6; 3.4	0.4
	Indoor with people	46 (209/449)	1.9	0.7; 5.2	0.2

Herd size	Base: <5 cattle				
	<10 cattle	39 (176/450)	1.5	0.8; 2.9	0.2
	>10 cattle	22 (99/450)	1.5	0.6; 3.2	0.3

Presence of donkeys		25 (112/450)	1.3	0.7; 2.3	0.4
Presence of oxen		80 (357/450)	0.8	0.4; 1.7	0.6
Presence of camels		2 (10/450)	1.7	0.2; 14.7	0.6
Not vaccinated cattle		20.4 (92/450)	1.2	0.6; 2.5	0.6
Contact with wildlife		19 (86/450)	0.9	0.4; 1.8	0.7
Not own bull for reproduction		54 (216/400)	1.1	0.6; 2.2	0.7
Human TB cases		19 (86/449)	1.1	0.6; 2	0.7
Presence of horses		9.3 (42/450)	1.3	0.4; 4.3	0.7
Presence of adult breeders (<10 years)		90.4 (407/450)	1.1	0.4; 2.9	0.8
Presence of calves (<1 year)		59.5 (268/450)	1	0.6; 1.8	0.9
Presence of juveniles (1–3 years)		61 (274/450)	1	0.5; 1.7	0.9
Presence of goats		31 (141/450)	1	0.6; 1.8	0.9

**Table 3:** Multivariable analysis of potential risk factors for positive cattle reactors using GLMM with kebele as random effect.

Variable	OR	95%CI OR	*p*-value
Purchase	1.5	0.9; 2.7	0.1
De-worming	1.8	0.8; 3.9	0.1
Communal grazing	1.3	0.7; 2.3	0.4
Other stock	1.7	0.8; 3.5	0.1

**Table 4:** Univariable analysis for risk factors for perceived TB cases in humans using GLMM with kebele as random effect.

Risk factor		Proportion% (Nb/Total)	OR	95% CI for OR	*p-*value
Cattle housing at night	Base: free-roaming				
	Outside shed	11 (48/449)	1.7	0.7; 3.9	0.4
	Indoor with people	46 (209/449)	1	0.4; 2.6	0.2

Raw milk consumption		68.5 (307/448)	0.3	0.5; 1.8	0.7
Raw meat consumption		74.4 (334/449)	1.1	0.6; 2	0.6
Keeping other livestock		70 (313/449)	1	0.6; 1.8	0.8

Cattle herd size	Base: <5 animals				0.2
	<10 animals	33.6 (151/449)	1.4	0.8; 2.5	
	<20 animals	13.6 (61/449)	1.5	0.7; 3.3	
	>20 animals	2.9 (13/449)	0.3	0.03; 2.8	

Number of cattle reactors	Base: none				0.8
	1	11.5 (52/450)	1.2	0.6; 2.4	
	2	0.9 (4/450)	1.4	7.2; 14.9	

Shepherding	Base: mixed shepherding				
	Children	36.3 (94/259)	0.7	0.3; 1.7	0.4
	Adult	13 (33/259)	1.7	0.6; 4.4	0.3

Continuous altitude					0.3
